# Antiplatelet Therapy for Atherothrombotic Disease in 2022—From Population to Patient-Centered Approaches

**DOI:** 10.3389/fcvm.2022.805525

**Published:** 2022-01-28

**Authors:** Georges Jourdi, Anne Godier, Marie Lordkipanidzé, Guillaume Marquis-Gravel, Pascale Gaussem

**Affiliations:** ^1^Research Center, Montreal Heart Institute, Montreal, QC, Canada; ^2^Faculty of Pharmacy, Université de Montréal, Montreal, QC, Canada; ^3^Université de Paris, Innovative Therapies in Haemostasis, INSERM UMR_S1140, Paris, France; ^4^Department of Anesthesiology and Critical Care, AP-HP, Université de Paris, Hôpital Européen Georges Pompidou, Paris, France; ^5^Faculty of Medicine, Université de Montréal, Montreal, QC, Canada; ^6^Service d'Hématologie Biologique, AP-HP, Université de Paris, Hôpital Européen Georges Pompidou, Paris, France

**Keywords:** platelets, aspirin, P2Y_12_ receptor antagonists, cardiovascular disease, precision medicine, bleeding, surgery

## Abstract

Antiplatelet agents, with aspirin and P2Y_12_ receptor antagonists as major key molecules, are currently the cornerstone of pharmacological treatment of atherothrombotic events including a variety of cardio- and cerebro-vascular as well as peripheral artery diseases. Over the last decades, significant changes have been made to antiplatelet therapeutic and prophylactic strategies. The shift from a population-based approach to patient-centered precision medicine requires greater awareness of individual risks and benefits associated with the different antiplatelet strategies, so that the right patient gets the right therapy at the right time. In this review, we present the currently available antiplatelet agents, outline different management strategies, particularly in case of bleeding or in perioperative setting, and develop the concept of high on-treatment platelet reactivity and the steps toward person-centered precision medicine aiming to optimize patient care.

## Introduction

Antiplatelet therapy, mainly including aspirin (acetylsalicylic acid, ASA) and P2Y_12_ receptor antagonists, is one of the most prescribed therapies in medicine due to the worldwide high prevalence of cardiovascular diseases (CVD) (1). Antiplatelet agents have significantly improved patient clinical outcomes during the last century, thus preventing a substantial number of atherothrombotic events and decreasing cardiovascular mortality rates. However, secondary bleeding complications remain relatively frequent (2–5). Substantial efforts have been made to develop tools to predict individual ischemic and bleeding risks, to minimize antiplatelet exposure among patients with high bleeding risk and/or low ischemic risk, and to improve percutaneous stent technologies reducing late thrombotic risks. This manuscript provides an overview of the antiplatelet agents currently available, details their management in clinical scenarios such as surgeries and bleeding complications, discusses the consequences of residual high on-treatment platelet reactivity (HTPR), and summarizes the current trends toward patient-centered precision medicine.

## Current Arsenal of Antiplatelet Agents

**Figure 1:**
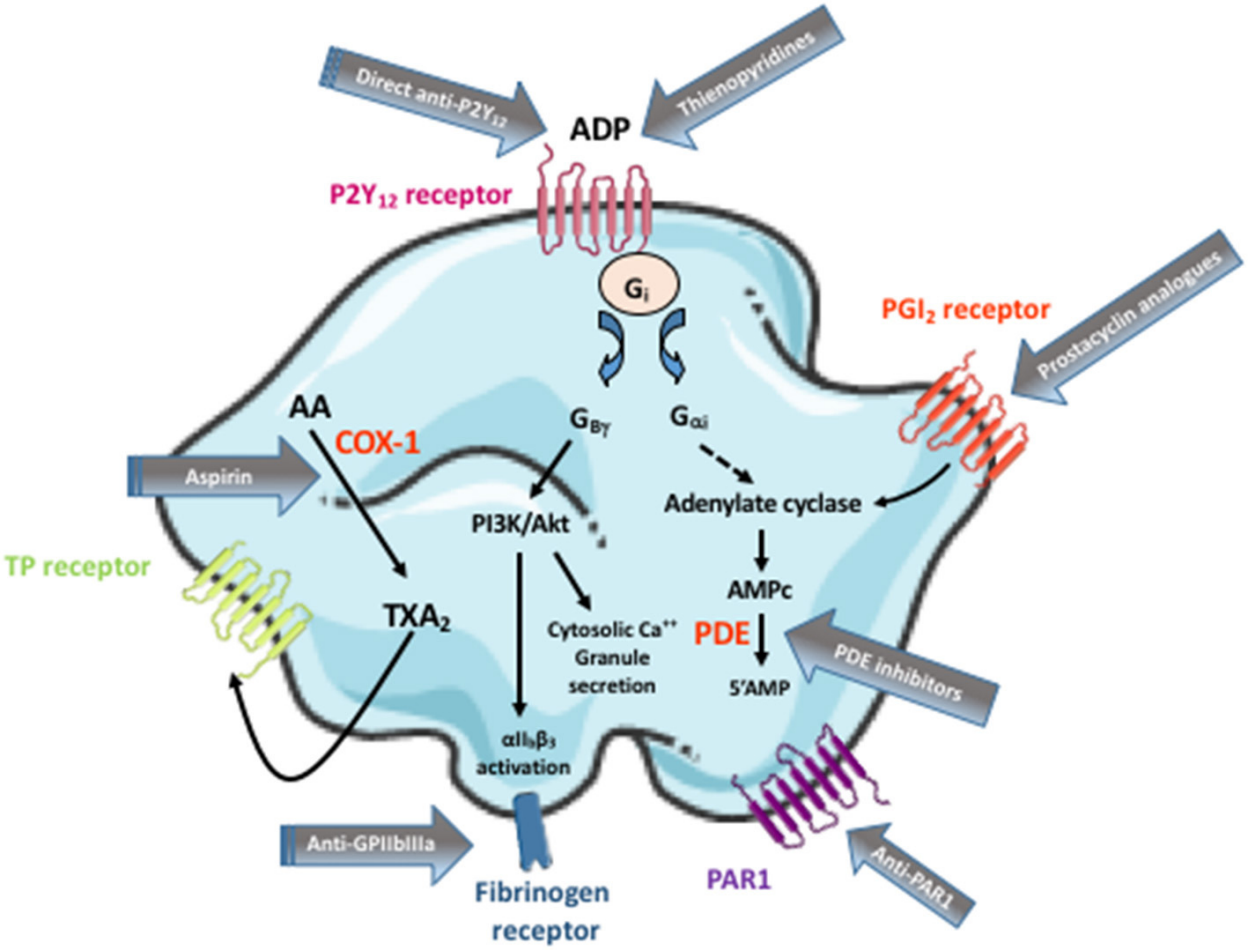
Targets of the commercialized antiplatelet agents. Arachidonic acid (AA) is produced by membrane phospholipids upon the action of phospholipase A_2_. It is metabolized in cyclic endoperoxydes by the cycloxygenase-1 (COX-1) enzyme, then in thromboxane A_2_ (TXA_2_) by the thromboxane synthase. TXA_2_ activates the Thromboxane Prostanoid (TP) receptor in return. ADP, by activating P2Y_12_ receptor, induces an inhibition of adenylate cyclase which downregulates cAMP (a powerful platelet inhibitor) synthesis. It also stimulates the phosphoinositide 3-kinase (PI3K) *via* G_βγ_ protein complex resulting in Akt stimulation, which activates a number of downstream substrate proteins thereby increasing the cytosolic Ca^2+^ levels and inducing granule secretion. Inversely, prostacyclin (PGI2) binds to its receptor on platelet surface and increases cAMP intraplatelet level. cAMP is metabolized by phosphodiesterases (PDE) in 5'AMP. Blocking ADP binding site with a P2Y_12_ receptor antagonist (including thienopyridines and direct anti-P2Y_12_), stimulating PGI_2_ receptor or inhibiting PDE maintains cAMP intraplatelet concentration at a high level thus keeping platelets in a resting state. Following coagulation activation, thrombin is generated and cleaves its receptor on platelet surface, i.e., the protease-activated receptor 1 (PAR1), resulting in its activation. TP, P2Y_12_, or PAR1 activation leads to a conformational change of the glycoprotein (GP)IIbIIIa (also called the integrin αII_b_β_3_) on platelet surface which links fibrinogen resulting in platelet aggregation. This figure does not aim to represent platelet physiology with the different signaling pathways. It rather illustrates in a very simple manner the targets of the currently available antiplatelet drugs.

Antiplatelet drugs represent key components of antithrombotic agents, mainly prescribed for the treatment and prevention of atherothrombotic diseases including acute coronary syndromes (ACS), stable coronary artery disease (CAD), peripheral artery disease (PAD), ischemic stroke, and transient ischemic attack (TIA). Antiplatelet agents act either by preventing the formation of second messengers, by interacting with intracellular signaling pathways, by blocking membrane receptors, or by inhibiting platelet aggregation *per se* (Figure 1). Their main pharmacokinetic/pharmacodynamic characteristics are summarized in Tables 1, 2.

**Table 1:** Pharmacological characteristics of oral antiplatelet drugs (6–21).

**Molecule**	**Mechanism of action**	**Drug class**	**Bioavailability**	**Elimination half-life**	**Onset of action after loading dose**	**Time to steady state platelet inhibition after maintenance dose**	**Time to platelet function recovery after drug cessation**
ASA	Acetylation of COX-1	COX-1 inhibitor	>40%^*^	15–20 min	~20 min	1 day	5–7 days
Clopidogrel	Irreversible P2Y_12_ antagonist	Thienopyridine	>50%	30 min^#^	2–6 h	5 days	7 days
Prasugrel	Irreversible P2Y_12_ antagonist	Thienopyridine	>78%	30–60 min^#^	30 min	3 days	7–10 days
Ticagrelor	Reversible P2Y_12_ antagonist	Cyclopentyl-triazolopyrimidine	36%	7–9 h	30 min	<5 days	3–5 days
Vorapaxar	Reversible PAR1 antagonist	PAR1 inhibitor	98%	5–13 days	–^‡^	21 days	4–8 weeks
Cilostazol	prevention of cAMP degradation	PDE3A inhibitor	Unknown	11–13 h	–^‡^	4 days	12–16 h
Dipyridamole^&^	prevention of cAMP degradation	PDE3 and PDE5 inhibitor	70%	13.6 h	–^‡^	4–7 days	–

*cAMP, cyclic adenosine 3′,5′-monophosphate; COX, cyclooxygenase; PAR,protease-activated receptor; PDE, phosphodiesterase*.

* *With a lower bioavailability with enteric-coated tablets in comparison to regular or chewable tablets*.

# *Active metabolite*.

& *Extended-release formulation*.

‡ *This antiplatelet drug is not administered at a loading dose*.

**Table 2:** Pharmacological characteristics of intravenous antiplatelet drugs (11, 13, 15, 19–23).

**Molecule**	**Mechanism of action**	**Drug class**	**Elimination half-life**	**Time to steady state platelet inhibition after maintenance dose**	**Time to platelet function recovery after drug cessation**
ASA	Acetylation of COX-1	COX-1 inhibitor	15–20 min	Few minutes	5–7 days
Cangrelor	Reversible P2Y_12_ antagonist	Adenosine triphosphate analog	3–6 min	≤5 min	30–60 min
Iloprost	Prostacyclin analog	Agonist of prostacyclin receptor	30 min	10–20 min	2 h
Eptifibatide	Reversible GPIIbIIIa inhibitor	Cyclic hexapeptide	2.5 h	≤15 min	4–8 h
Tirofiban	Reversible GPIIbIIIa inhibitor	Peptidomimetic	2 h	20–40 min	4–8 h

*GP, glycroprotein; mAb, monoclonal antibody*.

### Aspirin

ASA reduces the formation of thrombi *via* irreversible cyclooxygenase (COX)-1 inhibition, thereby suppressing platelet thromboxane A_2_ (TXA_2_) synthesis (6). It can be administered intravenously (in Europe) or as an oral loading dose (usually with chewable tablets in North America), in the first phase of ACS treatment, followed by daily maintenance dose, usually with enteric-coated tablets that may be absorbed more slowly and less efficiently in some patients (24). Lysine acetylsalicylate is the only formulation available in some countries that can be administered intravenously. Intravenous lysine acetylsalicylate provided more rapid and consistent platelet inhibition (evaluated by arachidonic acid-induced platelet aggregation measured using light transmission aggregometry) than oral ASA within the first hour after dosing in healthy volunteers (25). In the ECCLIPSE trial, a loading dose of intravenous lysine acetylsalicylate achieved an earlier platelet inhibition with less inter-individual variability than the oral loading dose of ASA (26). However, it has been suggested by some investigators that IV administration of lysine acetylsalicylate may have an acutely negative effect on endothelial vasodilatory prostaglandin production; the clinical impact of this potential endothelial inhibition has not been directly studied in clinical studies. Lysine acetylsalicylate can also be given orally, and was shown to induce fewer gastrointestinal adverse effects than ASA (27) with similar or higher inhibitory effect on light transmission platelet aggregometry in healthy volunteers and CAD patients (28, 29). Considering the limited evidence comparing the effects of intravenous lysine acetylsalicylate and oral ASA on platelet inhibition and endothelial prostacyclin biosynthesis in humans, this remains to be more extensively explored in future clinical studies.

In ACS setting, ASA is indicated in association with a P2Y_12_ receptor antagonist for 6–12 months depending on the balance between bleeding and ischemic risks (30–32). Dual antiplatelet therapy (DAPT) duration can be extended for up to 3 years in patients at high risk of ischemic events. Afterwards, ASA is recommended indefinitely as a single antiplatelet therapy (SAPT). ASA is also commonly prescribed in patients with stable CAD. It can be associated with clopidogrel for up to 12 months in patients undergoing elective coronary percutaneous intervention (PCI) (31–33). In patients with chronic symptomatic PAD, ASA is commonly prescribed as a long-term SAPT (34, 35). Its efficacy is counterbalanced by concerns of safety thus it is not recommended routinely in primary prevention, but can be considered for higher-risk patients on a case-by-case basis (36–40). ASA can also be prescribed in combination to clopidogrel for up to 90 days in patients with recent (within 30 days) TIA or stroke (41). It can also be prescribed for the secondary long-term prevention of stroke and TIA as a single therapy or in combination with dipyridamole (42, 43).

### P2Y_12_ Receptor Antagonists

**Figure 2:**
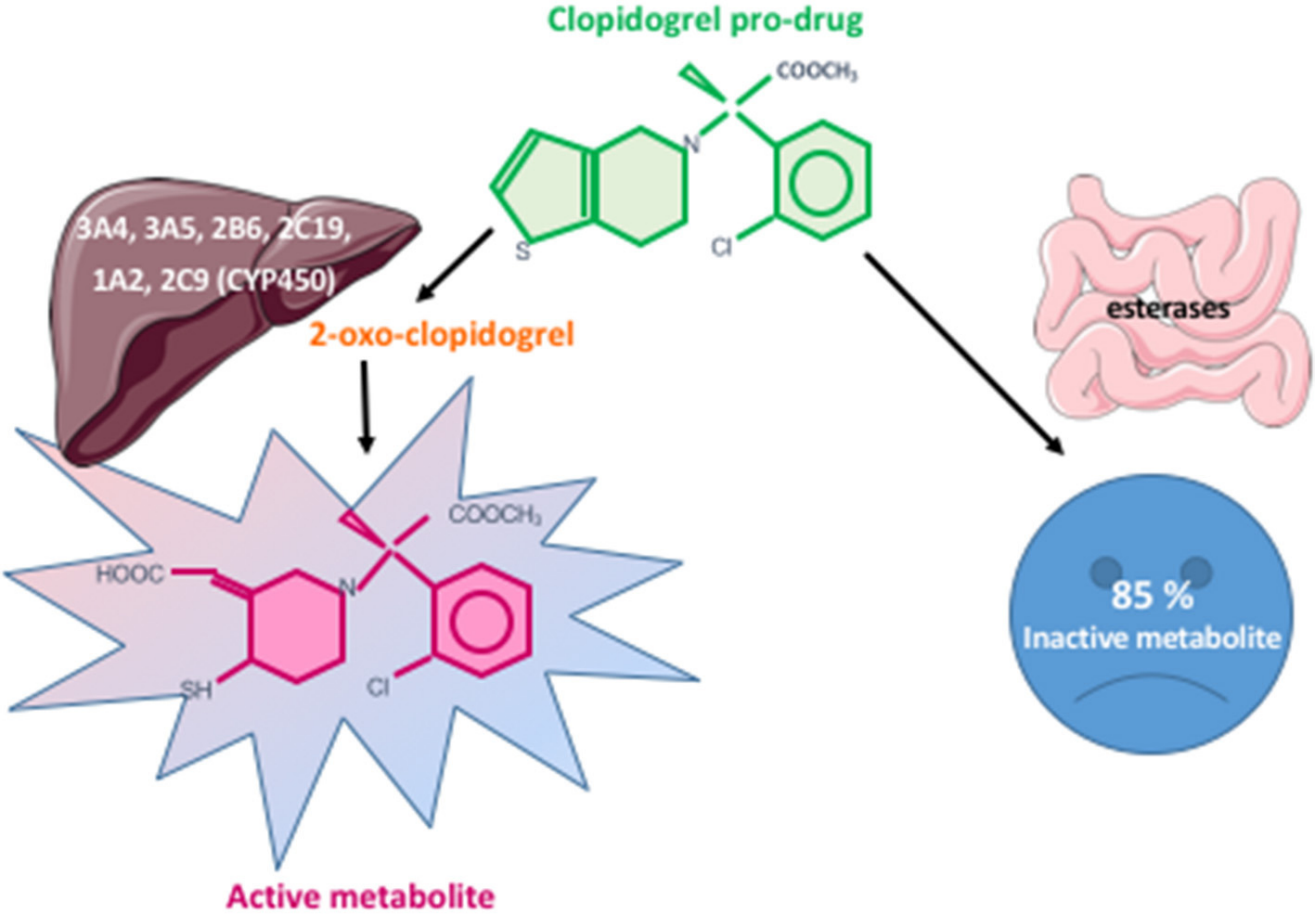
Clopidogrel metabolism pathways. Clopidogrel is a pro-drug. Eighty-five percent of the administered dose is metabolized into an inactive metabolite by intestinal esterases. The remaining 15% undergoes two sequential oxidative reactions involving several CYP enzymes leading, respectively, to 2-oxo-clopidogrel then to the active metabolite.

The P2Y_12_ receptor antagonists include two drug classes: the pro-drugs thienopyridines and the direct acting nucleoside–nucleotide derivatives. Clopidogrel is a pro-drug that requires two sequential oxidative reactions involving several CYP enzymes, mainly CYP2C19, to generate the active metabolite (Figure 2). Prasugrel is also a pro-drug. Its active metabolite irreversibly and competitively inhibits adenosine diphosphate (ADP)-induced platelet aggregation faster, more consistently and to a higher degree than clopidogrel (44, 45). Ticagrelor and cangrelor belong to the class of reversible P2Y_12_ receptor antagonists. While cangrelor is administered intravenously with a binding site at the P2Y_12_ receptor level not precisely defined (46), ticagrelor is an oral adenosine triphosphate (ATP) analog that binds the P2Y_12_ receptor at a distinct site from that of ADP. It does not require metabolic activation and achieves a faster, more potent and more predictable antiplatelet effect than clopidogrel (7). Very few studies compared prasugrel to ticagrelor antiplatelet effects. In diabetes mellitus (DM) patients with ACS, ticagrelor achieved a significantly higher platelet inhibition than prasugrel in Alexopoulos et al. (47) study while no difference was observed in Ndrepepa et al. (48) study. In ST elevation myocardial infarction (STEMI) patients undergoing primary PCI, ticagrelor did not show superiority compared to prasugrel in reducing platelet reactivity during the first 24 h (49). A recent meta-analysis compared the effects of prasugrel and ticagrelor on HTPR and low on-treatment platelet reactivity (LTPR) ACS patients. Prasugrel seemed less efficient in lessening the number of HTPR patients (50). However, this should be interpreted with caution since it was only obtained in a limited number of observational studies and case reports. In the light of these few studies (non-exhaustive list), no clear difference on platelet reactivity is reported between prasugrel and ticagrelor.

Ticagrelor is metabolized mainly by CYP3A4 to an active metabolite that represents 30–40% of circulating active drug. Additionally, ticagrelor inhibits adenosine reuptake *via* a membrane-bound channel called the type 1 equilibrative nucleoside transporter (ENT1) in erythrocytes and platelets, which might potentiate its antiplatelet effect (51). Ticagrelor is indicated in association with ASA in medically-managed ACS patients. Both prasugrel and ticagrelor are preferred over clopidogrel in ACS patients undergoing PCI, but prasugrel is contra-indicated in patients with a prior stroke or TIA. Prasugrel is associated with a significantly lower risk of CV events compared with ticagrelor in ACS patients planned for invasive therapy (52). It could be preferred over ticagrelor post-PCI in non-ST elevation (NSTE) ACS patients (53). Clopidogrel may be a favorable alternative to ticagrelor or prasugrel in NSTE-ACS patients aged 70 years or older because of the higher bleeding risk (54). A recent meta-analysis of six trials (DACAB, GLASSY, SMART-CHOICE, STOP-DAPT 2, TICO, and TWILIGHT) revealed that P2Y_12_ receptor antagonist monotherapy was associated with a similar risk of fatal and ischemic events and lower rates of major bleeding compared with DAPT in patients undergoing coronary revascularization particularly among females. ASA could thus be stopped 1–3 months after coronary revascularization and P2Y_12_ receptor antagonist monotherapy continued instead of DAPT, especially in women (55). However, no formal proposal has yet been published. In ACS patients undergoing urgent PCI and not pre-treated with an oral P2Y_12_ receptor antagonist, cangrelor might be an interesting therapeutic option (56). It can also be used in those who require DAPT bridging before surgery (57). Clopidogrel can also be prescribed as part of triple antithrombotic therapy [in association with ASA and an oral anticoagulant (OAC)] (31, 58, 59) in patients with atrial fibrillation suffering from ACS. Triple therapy is used during index hospitalization or up to 1 or 6 months (depending on patient ischemic and bleeding risks). It is followed by dual antithrombotic therapy (P2Y_12_ receptor antagonist plus OAC) for 1 year after coronary stenting then by an OAC indefinitely. While clopidogrel is commonly prescribed as part of the dual antithrombotic therapy, prasugrel is allowed in the Japanese guidelines (60) and ticagrelor might be an alternative to clopidogrel in patients with high ischemic and low bleeding risks according to the American and European guidelines (61, 62). In patients with mechanical heart valves undergoing PCI, a daily dose of clopidogrel in addition to vitamin K antagonist is indicated following an initial period of triple therapy (up to 6 months) (63). Prasugrel and ticagrelor are preferred over clopidogrel in DM patients with CVD necessitating DAPT, due to increased platelet reactivity seen at baseline and on-treatment in diabetic patients (4, 64–67).

Clopidogrel is the only P2Y_12_ receptor antagonist approved in association with ASA for 3–6 months in CAD patients undergoing elective PCI (31–33). It is also a good alternative for stroke and TIA prevention in patients with frequent headaches secondary to ASA/dipyridamole combination (43, 68).

### GPIIbIIIa Inhibitors

GPIIbIIIa inhibitors are intravenous antiplatelet agents that block the association of fibrinogen and von Willebrand factor (VWF) to the glycoproteins (GP) on the platelet surface. Abciximab was the first agent of this class, but it was withdrawn from the pharmaceutical market in 2019 following the interruption of its production by Janssen laboratories. Tirofiban is a non-peptide derivative of tyrosine mimicking the fibrinogen binding sequence within GPIIbIIIa while eptifibatide is a cyclic heptapeptide. Both are small molecules that inhibit GPIIbIIIa in a competitive manner with a stoichiometric ratio > 100:1 (69, 70). They are currently mainly considered as a bailout therapy in the event of angiographic evidence of a large thrombus, slow- or no-reflow, and other thrombotic complications in STEMI patients undergoing PCI or in NSTE-ACS patients undergoing high-risk PCI without pre-treatment with oral P2Y_12_ receptor antagonists (33).

### PAR1 Antagonist

Vorapaxar is an oral reversible PAR1 antagonist (71) that was approved by the Food and Drug Administration (FDA) in 2014. It has not yet gained the European Medicines Agency (EMA) approval (8). It is very rarely prescribed on top of standard antiplatelet therapy for secondary prevention in patients with a history of myocardial infarction or symptomatic PAD without any history of stroke, TIA or intracranial hemorrhage (72–74).

### Other Agents: Phosphodiesterase Inhibitors and Analog of Prostacyclin

Iloprost is a stable analog of prostacyclin (PGI_2_) that activates adelylate cyclase to increase intraplatelet cAMP level. It is also an arterial vasodilator which increases its therapeutic value for systemic administration in patients with severe PAD but increases the risk of hypotension (75). Dipyridamole is another antiplatelet agent that increases cAMP level within platelets by inhibiting its degradation by phosphodiesterase (PDE)3 and PDE5 (9, 76). It also induces endothelial synthesis and release of PGI_2_ (77, 78) and raises the extracellular levels of adenosine by inhibiting its reuptake by red blood cells and scavenges peroxy radicals, thus preventing vascular and tissue damage (76). It is worth mentioning that anticipated pharmacodynamics of both iloprost and dipyridamole should strictly match their pharmacokinetics. Dipyridamole is usually used in association with ASA for the secondary long-term prevention of stroke and TIA as previously mentioned.

Cilostazol is a selective inhibitor of PDE3A (the main subtype of PDE3 expressed in platelets) preventing the degradation of cyclic adenosine 3′,5′-monophosphate (cAMP) and to a lesser degree of cyclic guanosine 3′,5′-monophosphate (cGMP) thus resulting in an increase in the active forms of protein kinase A (PKA) and PKG. It also inhibits adenosine uptake and has a vasodilatory effect by relaxing the vascular smooth muscle cells (10). Cilostazol is recommended for the treatment of patients with intermittent claudication in the absence of tissue necrosis or rest pain (10). In the light of CSPS, CSPS2 and CASISP trials (79–81), it may also be used for secondary stroke prevention, particularly in Asian patients (82). Randomized trials are still needed to determine its usefulness for the secondary stroke prevention in non-Asian populations.

## Switching Between Antiplatelet Agents

**Figure 3:**
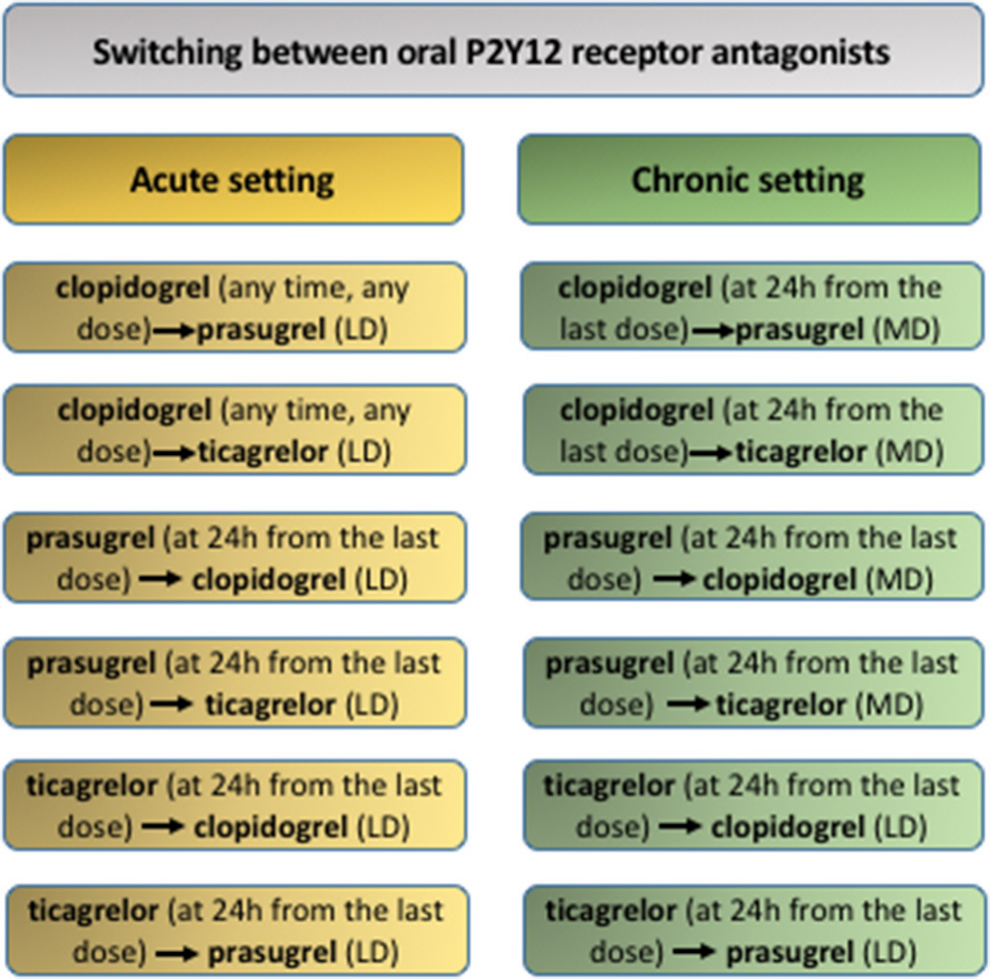
Switching strategy between oral P2Y_12_ receptor antagonists. LD, loading dose; clopidogrel LD = 600 mg; prasugrel LD = 60 mg; ticagrelor LD = 180 mg. MD, maintenance dose; clopidogrel MD = 75 mg q.d.; prasugrel MD = 10 mg q.d.; ticagrelor MD = 90 mg b.i.d.

Switching strategies between oral P2Y_12_ depends on the clinical setting (Figure 3). In the acute setting, switching to prasugrel or ticagrelor can occur irrespective of prior clopidogrel dosing and timing, whereas deescalation to clopidogrel should occur at 24 h from the last prasugrel or ticagrelor dose. Transitions between prasugrel and ticagrelor should also occur at 24 h from the last dose (46, 83) except in the Canadian guidelines, which advise a transition from ticagrelor to prasugrel 12 h after the last dose of the former in ACS setting (58). In the chronic setting, a loading dose is recommended at 24 h from the last dose when transitioning from ticagrelor to prasugrel or clopidogrel to avoid drug-to-drug interactions limiting the antiplatelet effect (46, 83).

Transition from cangrelor to oral P2Y_12_ receptor antagonists requires loading doses of clopidogrel and prasugrel to be administered immediately after the end of the cangrelor infusion to avoid drug interactions (46). Indeed, cangrelor blocks the binding of thienopyridine active metabolites on P2Y_12_ receptor, impairing their antiplatelet effect (83). On the contrary, ticagrelor can be administered before, during or after the cangrelor infusion (ideally within the last hour of the cangrelor infusion) without significant drug interactions (84).

## Peri-Operative Management of Antiplatelet Agents

Each year, 4–8% of patients receiving long-term antiplatelet therapy require major surgery (85). Peri-operative management of antiplatelet drugs is challenging since their continuation increases the risk of peri-procedural bleeding while their discontinuation increases the risk of thrombotic events. Moreover, delaying surgery can be detrimental in many cases including malignant and vascular diseases. Several factors should therefore be considered depending on whether the surgery is elective, urgent, associated with a high or low bleeding risk, or necessitates neuraxial anesthesia.

### Elective Surgery

The management of antiplatelet drugs is based on their indication and the procedure, particularly whether it is an elective cardiac or non-cardiac surgery. The risk of bleeding related to non-cardiac procedure can be divided into high, moderate, and low categories depending on the possibility of performing the procedure in patients receiving antiplatelet agents (none, SAPT or DAPT). Evaluation of the procedure-related bleeding risk also includes the type of anesthetic technique selected. POISE-2 study has suggested that administration of ASA before surgery and throughout the early postsurgical period had no significant effect on the rate of death or non-fatal myocardial infarction but increased the risk of major bleeding (86). However, continuation of ASA therapy is in general advocated in patients having stent. It could also be continued around the time of most elective non-cardiac surgeries since there are not many high bleeding risk procedures except complex hepato-biliary surgeries, open thoracic and thoraco-abdominal vascular surgeries, spinal, and intracranial surgeries (85, 87). Otherwise, a joint-decision making with the patient is suggested depending on baseline ischemic and bleeding risk. If the procedure requires discontinuation of antiplatelet therapy, the last intake of ASA, clopidogrel, ticagrelor, and prasugrel 3, 5, 5, and 7 days before surgery, respectively, is proposed (Table 3) (88). In case of intracranial surgery, 2 additional days free of antiplatelet therapy should be considered.

**Table 3:** Perioperative management of antiplatelet drugs in case of elective non-cardiac surgery.

**Molecule**	**Postponement of elective surgery**	**Resumption after surgery**	**Postponement of intracranial neurosurgery**
ASA	3–5 days^*^	24–96 h^*^	5 days
Clopidogrel	5 days	24–96 h	7 days
Prasugrel	7 days	24–96 h	9 days
Ticagrelor	5 days	24–96 h	7 days
Cangrelor	1 h	24–96 h^¶^	1 h

* *Whenever interrupted. ASA could be continued around the time of most elective surgeries*.

¶ *Resumption is usually performed with an oral P2Y_12_ receptor antagonist*.

The thrombotic risk associated with discontinuation should be assessed according to each specific indication of antiplatelet therapy: the risk is lower for patients receiving SAPT for CV prevention, for secondary stroke prevention or for lower extremity arterial disease than for those receiving DAPT following PCI (89). The risk is even higher for patients with a recent history of myocardial infarction or stent implantation as non-cardiac surgery exposes to an increased risk of stent thrombosis during the first few weeks, especially if both oral antiplatelet agents have to be discontinued due to a high procedure bleeding risk (31, 90). Thus, for patients with stent, procedures should be postponed until completion of DAPT if possible, and at least 1 month after implantation of drug-eluting stent (DES) when surgery should be rapidly performed (31, 58, 90, 91). If surgery is performed within the first month after DES implantation anyway, preoperative bridging therapy could be considered, using cangrelor or GPIIbIIIa inhibitors. GPIIbIIIa inhibitors should be started 2 days after stopping DAPT and stopped 6 h before surgery (12 h if creatinine clearance is <60 mL/min) (92), whereas cangrelor should be introduced 24 h after the last intake and stopped 1 h before the procedure. Resumption of P2Y_12_ receptor antagonists is recommended 24–96 h post-operatively and ideally within 48 h in patients with recent (<6 weeks) PCI or who presented with ACS (Table 3) (31).

ASA should be continued throughout the perioperative period in all patients requiring elective cardiac surgery. To minimize the risk of bleeding in patients under DAPT, a minimum interruption of ticagrelor and clopidogrel for 48–72 h (ideally for 5 days) and of prasugrel for 5 days (ideally for 7 days) is suggested in the Canadian guidelines (58). The European Society of Cardiology (ESC) and the Japanese Circulation Society (JCS) recommend a minimum interruption for 3, 5, and 7 days, respectively (33, 91). P2Y_12_ receptor antagonists should be resumed post-operatively as soon as it is deemed safe (32, 33, 58).

### Non-elective Invasive Surgery

In case of non-elective invasive procedure, management of oral antiplatelet drugs is based on their pharmacokinetic/pharmacodynamics parameters, the degree of urgency of the procedure and the associated bleeding risk. Platelet function testing may be considered to guide this management, but no evidence-based method has been approved for this indication (93, 94). When the bleeding risk induced by antiplatelet drugs may worsen the prognosis, measures should be taken to neutralize these drugs (95). ASA and thienopyridines, namely clopidogrel and prasugrel as well as their active metabolites, have short half-lives and bind irreversibly to their targets, thus exhibit inhibitory effects that last for the platelets' lifetime. Consequently, transfusion of a sufficient number of normal platelets should enable hemostasis by replacing the inhibited platelets (88). Administration of 0.7 × 10^11^ platelets/10 kg of body weight produces a rise in platelet count of ~40 × 10^9^/L (96). It is worth mentioning that the average size of a pool of platelets varies between countries and institutions. The above-mentioned dose is recommended in situations requiring neutralization of ASA while a higher dose is proposed for patients receiving clopidogrel or prasugrel (double for the former and even higher for the latter) (95). Neutralization of ticagrelor is more challenging due to its longer half-life along with that of its active metabolite compared to ASA, clopidogrel, and prasugrel. Consequently, circulating active compounds inhibit transfused platelets for at least 24 h after the last ticagrelor intake (97, 98). Beyond 24 h, platelet transfusion might be beneficial (88, 99). When the procedure must be performed within 24 h after the last intake, no therapeutic option could be recommended. When possible, postponing non-elective invasive procedures at least for a few hours or even a few days should be considered. Recombinant factor VIIa has been proposed to neutralize ticagrelor but its clinical efficacy has not been evaluated and it exposes to a thrombotic risk (100). Tranexamic acid is an option as it may reduce bleeding whether the patient has received antiplatelet drugs or not (101, 102). In April 2019, bentracimab (PB2452) received a breakthrough therapy designation from the FDA as a potential specific antidote for ticagrelor. It is a monoclonal antibody fragment that binds ticagrelor and its active metabolite with an affinity 100-fold higher than their affinities to P2Y_12_ receptor (103). It has completed a phase 1 clinical trial and further studies are awaited (104).

### Regional Anesthesia

Spinal epidural hematoma is a devastating complication of central neuraxial anesthesia, which includes spinal anesthesia and epidural with or without catheters. The risk of spinal hematoma related to ASA appears very low (105, 106), thus ASA is not a contraindication to neuraxial anesthesia if the benefit-risk ratio is favorable. The P2Y_12_ receptor antagonists carry a greater risk of bleeding than ASA, therefore neuraxial anesthesia is contraindicated in patients on clopidogrel, prasugrel or ticagrelor, unless those antiplatelet agents were discontinued 5, 7, and 5 days before the procedure, respectively, according to the French recommendations (88). In parallel, the American Society of Anesthesia and Regional Pain Medicine recommends a 5-days discontinuation period for all oral P2Y_12_ receptor antagonists (107). The insertion of an epidural catheter makes the management of antiplatelet agents more complex. Catheter manipulation and removal carry similar risks to insertion and the same rules should apply. The use of an epidural catheter should not compromise the postoperative resumption of antiplatelet agents, especially of P2Y_12_ receptor antagonists and DAPT. The benefit of the catheter should thus be carefully balanced with the thrombotic risk of delaying resumption (107).

Peripheral nerve blocks can be divided into two groups according to the degree of bleeding risk. Peripheral nerve blocks associated with a high bleeding risk follow the same rules as neuraxial anesthesia. They include deep blocks such as the para-sacral sciatic block, posterior lumbar plexus block, infraclavicular block, etc. On the contrary, peripheral nerve blocks associated with low bleeding risk could be performed in patients on SAPT or DAPT. Those blocks include superficial blocks such as the axillary block, popliteal sciatic block, femoral block, etc.

## Management of Bleeding Associated With Antiplatelet Therapy

Bleeding associated with antiplatelet drugs is heterogeneous thus no one course can be universally recommended. Several characteristics should be considered: type of bleeding (spontaneous vs. associated with trauma or injury), location and intensity of bleeding, type of antiplatelet therapy, time-interval since the last intake, etc. Combination of antiplatelet drugs or the use of the more-potent P2Y_12_ receptor antagonists (ticagrelor or prasugrel) are associated with an increased risk of hemorrhagic complications in comparison to ASA or clopidogrel monotherapy (5). In all cases, etiological and symptomatic treatment of bleeding is essential. Conventional hemostatic means include mechanical (embolization, endoscopy, compression, surgery, etc.) and resuscitation measures (fluids, red blood cells, plasma and/or factor concentrates administration, prevention of hypothermia, etc.). If hemostatic measures are not sufficient to stop the bleeding, neutralization of antiplatelet therapy could be considered taking into account the type of antiplatelet drug, the time-interval since the last intake, the ischemic risk of the patient and the characteristics of the bleeding event (site, severity). Practical guidelines for the bleeding management in patients on antiplatelet therapy are mainly based on expert opinion since no solid clinical evidence are available. They could be summarized as followed.

### Gastrointestinal Hemorrhage

Gastrointestinal hemorrhage is the most frequent bleeding complication associated with antiplatelet agents. Gastrointestinal bleeding secondary to ASA therapy is dose-related (42) most probably linked to the inhibition of COX-2 (and COX-1) in the endothelial cells lining the stomach, which suppresses cytoprotective PGE_2_ production. A meta-analysis of adverse events of low-dose ASA in 14 randomized controlled trials reported a modest annual absolute rate of 0.12%/year, slightly higher than that induced by clopidogrel (2, 108). Prasugrel and ticagrelor are associated with a higher rate compared to clopidogrel (3, 4). This rate is estimated at 1.3–4.6%/year with the DAPT combining ASA and clopidogrel (109). Endoscopic control of bleeding is recommended for patients suspected to have upper gastrointestinal hemorrhage (110). Proton pump inhibitors are recommended in all patients as they improve outcomes in acute bleeding and prevent upper gastro-intestinal re-bleeds in patients continuing SAPT or DAPT (111–113). Moreover, proton pump inhibitors are also recommended in patients with a history or an increased risk of gastrointestinal bleeding, including the elderly and patients with concomitant use of vitamin K antagonists, steroids, and nonsteroidal anti-inflammatory drugs. Nevertheless, their routine use for patients at low risk of gastrointestinal bleeding is not recommended (31, 32). The choice of the proton pump inhibitor should take into account the degree of inhibition of CYP2C19, particularly in patients receiving clopidogrel. Omeprazole has been shown to reduce platelet inhibition *ex vivo* in the OCLA study (114), however, no significant increase in CV events was noted in patients treated with clopidogrel and omeprazole in the COGENT trial (115). Pantoprazole and esomeprazole appear to be safe alternatives (116), while lansoprazole might impair platelet inhibition in patients receiving clopidogrel (117). In case of severe bleeding, the benefit of platelet transfusion has been poorly assessed (111, 118). It should thus be reserved to specific severe cases after failure of etiological and symptomatic treatments (95, 119).

The dilemma of if and when antiplatelet agents should be reintroduced following gastrointestinal bleeding persists since randomized trials are lacking. Consequently, practice is variable and not necessarily evidenced-based. As such, in patients under secondary prophylaxis, there is a clear benefit in restarting antiplatelet therapy. When the risk of re-bleeding is low, single agent ASA can be continued without interruption, especially when endoscopic control has been achieved. When bleeding risk is high, ASA should be withheld but reintroduced early (within 3–7 days) as outlined in the recent review by Scott et al. (120). For patients receiving DAPT, particularly following recent cardiac stent insertion, ASA should be continued and the reintroduction of the second antiplatelet agent should be discussed with the cardiologist (99).

### Intracranial Hemorrhage

Conflicting results were reported on the association between ASA use and the risk of intracranial hemorrhage or cerebral microbleeds (121–124). More information may be generated from the ongoing ASPREE-NEURO study (125). Prasugrel and ticagrelor were associated with more intracranial hemorrhage compared to clopidogrel in the TRITON-TIMI 38 and PLATO trials, respectively. Prior stroke or TIA and previous intracranial hemorrhage are contraindications to prasugrel. Ticagrelor should be used with precautions in this context (4, 7). Alike, vorapaxar is contraindicated in patients at high risk of intracranial hemorrhage (8). That said, once intracranial hemorrhage occurs, antiplatelet therapy worsens the prognosis.

Efficacy of platelet transfusion in this case depends on many factors including the type of the drug, the time from the last drug intake and from the hemorrhage, the site of bleeding and the mechanism (spontaneous vs. traumatic). The required dose and the optimal timing of delivery relative to the last dose of antiplatelet agent remain uncertain. Platelet transfusion is recommended in treated patients suffering from intracranial hemorrhage and requiring urgent neurosurgery (121). Guidelines also propose platelet function testing prior to transfusion when possible (95). In non-surgical settings, platelet transfusion efficacy is not recommended to neutralize ASA (121) since the PATCH study reported worsened outcomes post-transfusion in patients on ASA as monotherapy and presenting supratentorial intracerebral hemorrhage with Glasgow Coma Scores ≥ 8 on admission (126). No study has assessed yet the effect of platelet transfusion in patients presenting intracranial hemorrhage with altered consciousness or in cases of treatment by P2Y_12_ receptor antagonists, thus no formal recommendation could be proposed.

### Hemorrhagic Shock

Platelet function recovery is essential and critical in case of hemorrhagic shock in patients under antiplatelet therapy. Neutralization of antiplatelet drugs is therefore usually proposed (88, 127, 128).

### Non-severe Bleeding

Non-severe bleeding complications only require symptomatic treatment without neutralizing or discontinuing antiplatelet therapy, along with the re-evaluation of the indication for antithrombotic treatment.

## High On-Treatment Platelet Reactivity and Precision Medicine

Numerous studies have demonstrated substantial interpatient variability in the responsiveness to antiplatelet therapy, based on clinical outcomes and/or laboratory methods used to assess platelet reactivity. It is critically important to note that true pharmacological resistance, i.e., non-response, to antiplatelet therapy is extremely rare. In most cases, HTPR cannot be directly pinpointed to a pharmacological mechanism (such as a mutation of the drug targets or alteration of the prodrug bioactivation, etc., cf infra). Notwithstanding, high residual on-treatment platelet reactivity either on ASA or on DAPT is a negative prognostic factor for the occurrence of future CV events, once drug interactions and non-compliance have been ruled out. Similarly, LTPR is associated with increased risk of bleeding, making the selection of the intensity and duration of antiplatelet therapy for individual patients a clinical challenge. A tailored personalized therapeutic approach, particularly on the basis of a better stratification of the individual risk profile and the evolution in stent technology, in pharmacogenomics and in laboratory assays used to evaluate platelet reactivity, would be beneficial.

### Scores Predicting Individual Ischemic and Bleeding Risk

Many scores and risk stratifying models have been developed to help tailor antiplatelet therapy with the aim to maximize ischemic protection and minimize bleeding risk, although an increasing body of evidence suggests that ischemic and bleeding risks are dynamic, fluctuating in time, and depending on patient characteristics, including ethnicity. For instance, East Asian patients have a lower rate of ischemic events after PCI compared to Caucasian patients while their bleeding risk is higher (129). A standardized definition of high bleeding risk has been recently proposed by consensus by the Academic Research Consortium for high bleeding risk (ARC-HBR) and validated in ACS or CAD patients undergoing PCI. It includes advanced age, anemia, thrombocytopenia, liver cirrhosis, use of oral anticoagulation, steroids or non-steroidal anti-inflammatory drugs, chronic kidney disease, history of spontaneous bleeding, stroke or active malignancy, chronic bleeding diathesis, planned surgery and trauma or surgery within 30 days before PCI (130). The PRECISE-DAPT score helps predicting bleeding events at 1 year in patients having completed successful PCI and requiring DAPT. This score evaluates five items namely age, creatinine clearance, hemoglobin, white blood cell count, and history of bleeding (131). Very recently, Pelliccia et al. (132) showed that the risk of bleeding changes over time in a substantial proportion of patients on DAPT after PCI. Frequent evaluation and recalculation of the PRECISE-DAPT score might therefore offset the excess bleeding associated with long DAPT in patients with comorbidities. Four other scores, namely DAPT, PARIS, CALIBER, and CREDO Kyoto scores, stratify both ischemic and bleeding risks (133–136). Patients with acute activation of the atherosclerotic process (ACS, ischemic stroke or TIA, and acute limb ischemia), extended diffuse atherosclerotic disease (PAD, diffuse coronary atherosclerosis, aortic, and carotid plaques), high burden of risk factors (current smoking habit, DM, and systemic arterial hypertension) and/or comorbidities (low ejection fraction, i.e., <30%, heart congestive disease, previous myocardial infarction and previous PCI) are particularly at higher risk of ischemic complications (133). Several other risk assessment models have also been proposed in the TRA 2P-TIMI 50, ADAPT-DES, HORIZON AMI, TRILOGY ACS, and CRUSADE trials (137–142). Of note, these scores and risk-predicting models still need to be tested in prospective randomized controlled trials to verify their real predictive values in guiding antiplatelet therapy especially that their discrimination ability in retrospective validation studies is at best moderate-to-good (143, 144).

### Choice of Stent Type

Individualization of antiplatelet therapy should also consider the stent type. Thrombotic risk of any stent type is highest initially and decreases over time but is never null. Bare metal stents (BMS) were associated with high rate of repeat revascularization because of restenosis (145). First generation DES (eluting paclitaxel or sirolimus) were subsequently developed to reduce this risk. However, they are associated with a higher risk of late and very late in-stent thrombosis compared to BMS. Thus, long-term DAPT was recommended (146). Unlike bioresorbable scaffolds, second-generation DES (zotarolimus, ridaforlimus, and everolimus) confer a lesser in-stent thrombotic risk, concentrated during the first 30 days (31, 147) enabling reduction of the DAPT duration. Compared to the first-generation DES, they have less bulky struts, use less thrombogenic polymers and provide better kinetics of drug release which explains the widespread preference for second-generation DES over the others. Moreover, guiding stent implantation using meticulous intravascular images decreases the thrombotic risk (148). Continuous development in stent technology will undoubtedly provide new perspectives in the contemporary use of antiplatelet therapy. Further well-designed studies are necessary to explore safe antiplatelet scenarios with newer generation stents.

### Pharmacogenomics of Antiplatelet Therapy

Although the cause of the variability in antiplatelet therapy efficacy is likely to be multifactorial, a substantial part is attributed to genetic etiology. The study of pharmacogenomics presents the possibility of individualized optimization of antiplatelet therapy tailored to each patient's unique genetic traits. ASA and clopidogrel are the most studied antiplatelet agents regarding genetic polymorphisms. Some genetic variants in COX-1 and 2 proteins (*PTSG1* and *PTSG2* genes), in P2RY1 receptor gene, or in some platelet GP such as the integrin α2/β1 (GPIaIIa), a platelet collagen receptor encoded by *ITGA2* gene, or the IIIa subunit of GPIIbIIIa receptor (*ITGB3* gene) (149, 150) have been reported to be associated with HTPR. However, the clinical outcomes in patients carrying these variants have not robustly demonstrated lesser clinical benefit from ASA therapy.

The strongest genetic associations for platelet responses to clopidogrel are with the CYP responsible for its bioactivation, most notably CYP2C19. The pharmacological importance of CYP2C19 genotypes in clopidogrel therapy has been first described in healthy volunteers (151) then extensively studied in various patient groups. This enzyme is highly variable in the general population with at least eight alleles of various levels of activity that could be classified into 4 categories (152, 153): (i) extensive metabolizer having wild type fully functional allele (^*^1) with a prevalence of 69.5% in the general population; (ii) poor metabolizer carrying two loss-of-function alleles (^*^2–^*^9) with ^*^2 (rs4244285, c.681G>A) having a minor allele frequency (MAF) of ~12–15, 15–18, and 25–30% in European, African, and Asian populations, respectively; (iii) intermediate metabolizer carrying one loss-of-function allele plus one functional or increased-activity allele; and (iv) ultra-rapid metabolizer having at least one increased-activity allele [^*^17 (rs12248560, −608C>T) having a MAF of ~20% in European and Black populations and 5% in Asian populations] (152, 154, 155). These genetic variants might significantly affect clopidogrel metabolism thus its active metabolite plasma concentrations modifying therefore the risk of ischemic and bleeding complications (153, 156, 157). Indeed, poor active metabolite production and ensuing HTPR on clopidogrel can be overcome by increasing the dose of clopidogrel in heterozygous carriers of the loss-of-function allele (namely the CYP2C19^*^2) but not in homozygous carriers as was previously shown in the CLOVIS and CLOVIS-2 studies (158, 159).

Prasugrel and ticagrelor are sensible alternatives among patients with loss-of-function genetic variants since their pharmacokinetics, antiplatelet effects and clinical effectiveness are less, if at all, impacted by CYP2C19 genotype (156, 160). Of note, genetic variants associated with reduced clinical effectiveness of prasugrel and ticagrelor have not been identified to date. Similarly, while patients with increased CYP2C19 activity, such as ^*^17 homozygotes, have been shown to exhibit increased response to clopidogrel (161) a recent study failed to show any clinical impact of the ^*^17 variant in patients treated by clopidogrel (162).

Besides, genetic variants in *ABCB1* and CYP enzymes (CYP3A4^*^1G, CYP3A5^*^1, and CYP3A5^*^3) genes have been investigated for their impacts on clopidogrel pharmacokinetic profile, however, heterogeneous outcomes are reported and more studies are required to draw clear conclusions of their importance (163, 164).

Most genetic intervention studies have thus targeted the CYP2C19 polymorphism for personalized antiplatelet approaches, and some have shown promise, including PHARMCLO, POPular Genetics and TAILOR-PCI trials (157, 165, 166). While the recent ESC guidelines do not make recommendations regarding CYP2C19 genotyping (167), the American College of Cardiology/American Heart Association (ACC/AHA), and the Society for Cardiovascular Angiography and Interventions (ACCF/AHA/SCAI) PCI guidelines state that CYP2C19 genetic testing may be considered in patients undergoing PCI who are at high risk of poor outcome due to inadequate platelet inhibition (168). In the setting of acute ischemic stroke, TIA and cerebrovascular intervention, prospective data characterizing the impact of a CYP2C19 genotype-guided anti-P2Y_12_ therapy selection strategy on adverse neurological, vascular and bleeding outcomes are still needed. No recommendations regarding the use of CYP2C19 testing were issued either by the American Stroke Association (169) or the European Stroke Organization (170).

### Laboratory Assessment of Antiplatelet Therapy

While some trials suggested positive impact of personalized antiplatelet therapy based exclusively (i.e., without relying on pharmacogenomics data) on platelet function testing (MADONNA, ISAR-HPR, TROPICAL-ACS, CREATIVE, and Aradi et al.) (171–175), others have failed to show benefit (ARCTIC, ANTARCTIC, TRIGGER-PCI, and GRAVITAS) (176–179) (Table 4). Discrepancies could be explained, at least partly by study design, selected patient populations, timing of platelet function, antiplatelet therapy strategies, and evaluated assays. That said, platelet function testing in treated patients is currently not recommended in routine clinical practice by either ACC/AHA or ESC. However, it may be considered in selected patients at high ischemic risk leading to poor clinical outcomes or with variable therapeutic observance. According to the 2018 ESC guidelines for myocardial revascularization, de-escalation of P2Y_12_ receptor antagonists based therapy (e.g., from prasugrel to clopidogrel in patients with normal clopidogrel platelet inhibition response) guided by platelet function testing may be considered, particularly in ACS patients unsuitable for 12-month DAPT (33).

**Table 4:** Clinical trials evaluating personalized antiplatelet therapy based exclusively on platelet function testing.

**Study**	**Number of patients**	**Studied population**	**Antiplatelet therapy**	**Platelet function test**	**Outcome**
ANTARCTIC (176)	877	Post-PCI in ACS patients (≥75 years old)	ASA+prasugrel (5 mg)	VerifyNow^TM^	No improvement in clinical outcome
			LTPR: ASA+clopidogrel (75 mg)		
			HTPR: ASA+prasugrel (10 mg)		
Aradi et al. (173)	741	Post-PCI in high risk ACS patients	ASA+clopidogrel (75 mg)	Multiplate^®^	Unlike high-dose clopidogrel, switching to prasugrel reduces ischemic risk in HTPR patients
			HTPR: ASA+prasugrel (10 mg) or ASA+clopidogrel (150 mg or additional 600 mg LD+75 mg)		
ARCTIC (179)	1,227 (control)	Post-PCI in CAD/ACS patients	Control: treatment choice left to physician's discretion	VerifyNow^TM^	No improvement in clinical outcome in the guided-therapy group
	1,213 (guided)		HTPR: ASA+prasugrel (10 mg) or ASA+clopidogrel (150 mg)		
			LTPR: ASA+clopidogrel (75 mg)		
GRAVITAS (178)	2,214	Post-PCI in CAD/NSTE-ACS patients	ASA+clopidogrel (75 mg)	VerifyNow^TM^	No reduction of the incidence of death or cardiovascular events
			HTPR: ASA+clopidogrel (150 mg)		
ISAR-HPR (172)	428 (control)	Post-PCI in CAD/ACS patients	Control: ASA+clopidogrel (75 mg)	Multiplate^®^	Significant reduction of the incidence of death from any cause in the guided-therapy group
	571 (guided)		Guided: ASA+clopidogrel (75 mg) vs. ASA+clopidogrel (additional 600 mg LD+75 mg) or ASA+prasugrel (10 mg) if HPR		
MADONNA (171)	395 (control)	Post-PCI in STEMI and NSTE-ACS patients	Control: ASA+clopidogrel (75 mg)	Multiplate^®^	Reduction of the incidence of stent thrombosis and ACS in the guided-therapy group but no difference in cardiac death or bleeding
	403 (guided)		Guided: ASA+clopidogrel (75 mg) vs. ASA+clopidogrel (additional 600 mg LD + 75 mg) or ASA+prasugrel (60 mg LD + 10 mg) if HTPR		
TRIGGER-PCI (177)	236	Post-PCI in CAD HPR patients	ASA+clopidogrel (75 mg) vs. ASA+prasugrel (10 mg)	VerifyNow^TM^	Switching from clopidogrel to prasugrel afforded effective platelet inhibition.
					Study stopped prematurely for futility (lower than expected incidence of adverse ischemic events)
TROPICAL-ACS (174)	1,306 (control)	Post-PCI in ACS patients	Control: ASA+prasugrel (10 mg)	Multiplate^®^	Non-inferiority of guided therapy in terms of cardiovascular death, myocardial infarction, stroke or bleeding complications
	1,304 (guided)		Guided: ASA+clopidogrel (75 mg) vs. ASA+prasugrel (10 mg) if HTPR		

*ACS, acute coronary syndrome; CAD, stable coronary artery disease; DES, drug-eluting stent; HTPR, high on-treatment platelet reactivity; LD, loading dose; LTPR, low on-treatment platelet reactivity; NSTE, non-ST elevation; PCI, percutaneous coronary intervention, PFT, platelet function testing; STEMI, non-ST elevation myocardial infarction*.

**Figure 4:**
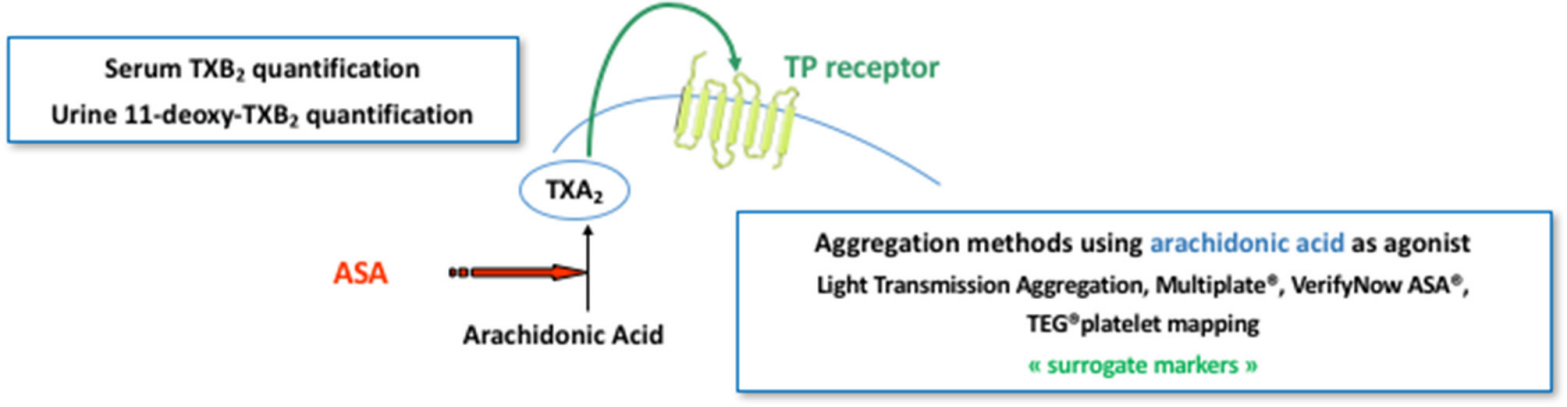
Pharmacodynamics of ASA. ASA pharmacodynamics can be evaluated using either an aggregation-based method evaluating the *in vitro* platelet capacity to be activated with arachidonic acid or by assessing the *in vivo* biosynthesis of thromboxane A_2_ *via* the quantification of its stable metabolite, namely thromboxane B_2_ (TXB_2_) in serum or 11-deoxy-TXB_2_ in urine. Aggregation-based method could be performed using the gold standard method for platelet function analysis, i.e., light transmission aggregometry, or one of the commercialized point-of-care tests, mainly Multiplate^®^, VerifyNow ASA^®^, and TEG^®^ platelet mapping.

**Figure 5:**
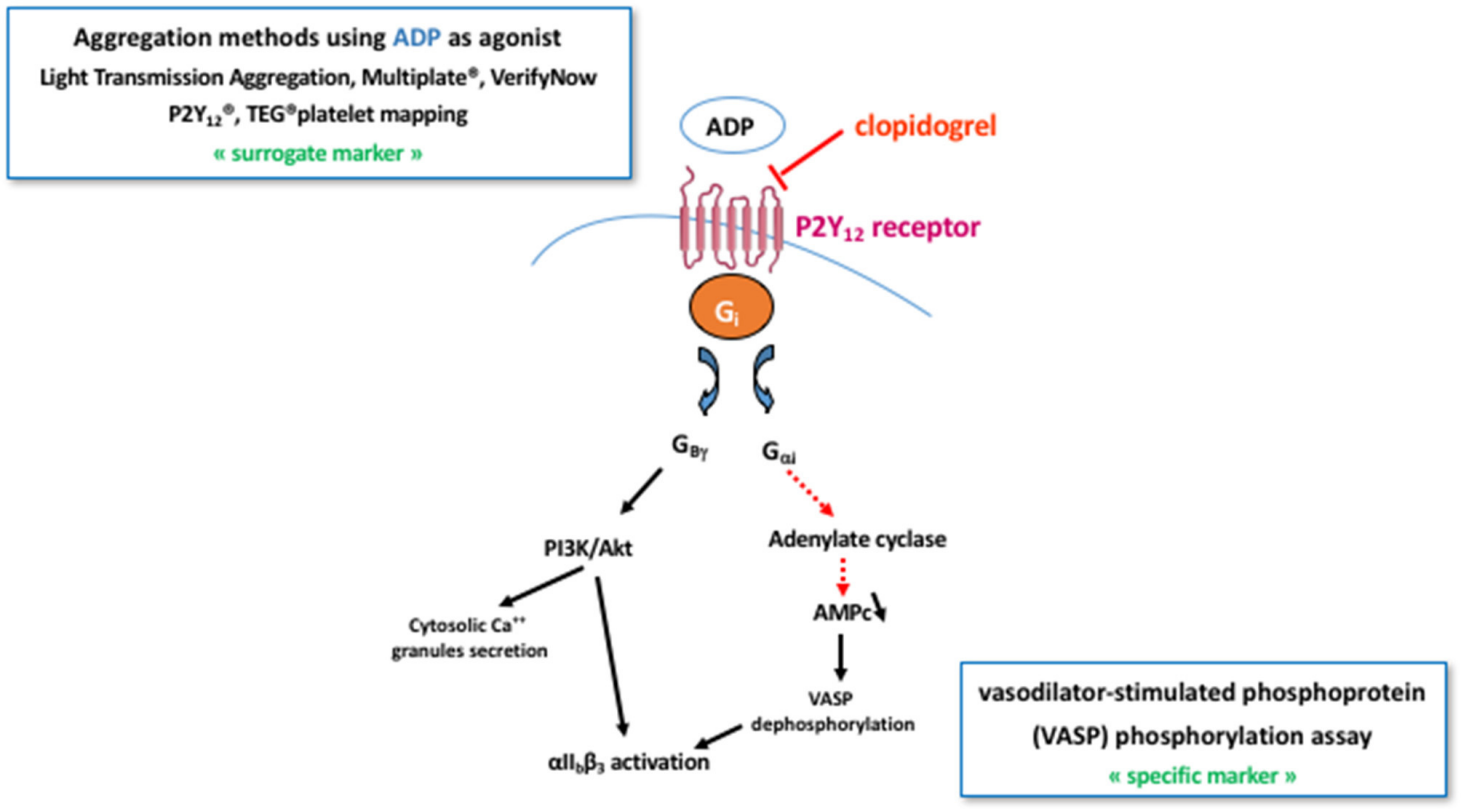
Pharmacodynamics of clopidogrel. Clopidogrel pharmacodynamics can be evaluated using either an aggregation-based method with ADP as platelet agonist or, more specifically by measuring the phosphorylation rate of the vasodilator-stimulated phosphoprotein (VASP) using flow cytometry or ELISA assays. The former can use the gold standard method for platelet function analysis, i.e. light transmission aggregometry, or one of the commercialized point-of-care tests, mainly Multiplate^®^, VerifyNow P2Y_12_, and TEG^®^ platelet mapping. PI3K, phosphoinositide 3-kinase.

The gold standard method for platelet function analysis is the light transmission aggregometry, a functional assay performed in citrate-anticoagulated platelet rich plasma, ideally within 4 h following blood sampling without any adjustment of the platelet count (unless it exceeds 600 × 10^9^ L^−1^) (180). Platelet aggregation induced by arachidonic acid and ADP might be used to assess ASA- and P2Y_12_ receptor antagonists-related inhibition with a maximal platelet aggregation ≤ 20% (181) and ≤ 50% (182) in good responders' patients, respectively. However, this assay is time-consuming, requires sample preparation and dedicated laboratory resources, is poorly standardized and lacks specificity. Indeed, ASA was shown to influence ADP-induced platelet aggregation and, inversely, clopidogrel significantly inhibited arachidonic acid-induced platelet aggregation in patients on DAPT (183). ASA antiplatelet efficacy can also be evaluated by measuring TXA_2_ stable metabolite concentration in serum, namely TXB_2_, or 11-deoxy-TXB_2_ in the urine (Figure 4). However, these tests are not adapted to emergency context and are not entirely specific to ASA antiplatelet effect since TXA_2_ might be synthetized and secreted by other cells than platelets on one hand, and anti-P2Y_12_ receptor antagonists as well as non-steroidal anti-inflammatory drugs and anticoagulants (by inhibiting thrombin generation thus subsequent platelet activation) can also inhibit the amplification of platelet activation thus the TXA_2_ biosynthesis on the other hand (183). The pharmacological inhibition of the P2Y_12_ receptor can be evaluated by measuring the phosphorylation rate of the vasodilator-stimulated phosphoprotein (VASP) using flow cytometry or ELISA assays, which is insensitive to concomitant ASA therapy (183) and can be performed on blood samples drawn with a time-frame of 48 h (Figure 5). This test has shown good correlations with plasma concentrations of the drugs and their active metabolites, particularly for clopidogrel (184) and ticagrelor (185). Therefore, while both being centered on and close to the molecular effect of the antiplatelet drugs, VASP assay is insensitive to ASA while the levels of TXB_2_ in serum might be affected by P2Y_12_ receptor antagonists in patients on DAPT.

Assessment of platelet function might be particularly helpful in some urgent situations such as in the perioperative context or in bleeding patients with missing information of their antiplatelet therapy such as in unconscious, mentally incapacitated or amnesic patients. Indeed, point-of-care (POC) tests can aid determination of safe timing of invasive procedures in treated patients and management of hemorrhagic complications although these tests still need refinement and standardization and special expertise is needed to reliably interpret the results (186, 187). Moreover, the majority of the interventional studies (if not all) that evaluated the impact of the personalized antiplatelet therapy based exclusively on platelet function testing (Table 4) included POC tests rather than the light transmission aggregometry with usually missing pre-analytical and analytical assay details. Commercialized POC platelet analyzers include PFA-100^®^, VerifyNow^TM^, Multiplate^®^, Quantra, and TEG^®^-PM (platelet mapping). Briefly, PFA-100^®^ is a quick test in which citrate-anticoagulated whole blood is aspirated in the presence of high shear rate through an aperture in a membrane coated with collagen and ADP or epinephrine until the aperture is completely occluded by a platelet plug. This POC is highly affected by VWF antigen level and activity and lacks sensitivity and reliability thus should not be used to assess antiplatelet therapy (188–190). VerifyNow^TM^ is a quick but relatively expensive assay which consists on activating thrombin receptors on platelets surface resulting in their agglutination with the fibrinogen-coated beads thus increasing the light transmission through the sample. Yet, the analytical details explaining how light can be transmitted though whole blood remains undisclosed. It can be effective to assess ASA, P2Y_12_ receptor antagonists and GPIIbIIIa inhibitors using three specific cartridges. It has been assessed in many clinical trials (176–179) and was shown to accurately reflect the plasma concentration of some active antiplatelet compounds (184, 185). Nevertheless, several factors influence its performance, including fibrinogen levels, hematocrit, platelet count, triglyceride levels, and time from blood sampling to testing (191). The Multiplate^®^ measures the increase of impedance between two electrodes caused by platelet aggregation in whole blood and is relatively sensitive to antiplatelet agents. Many studies demonstrated the benefit of tailoring antiplatelet therapy according to platelet function analysis using Multiplate^®^ (171–174, 192). However, this test requires more laboratory expertise and is more time-consuming than other bedside assays. Finally, TEG^®^-PM and Quantra are POCs used in surgery and anesthesiology that measure platelet inhibition relative to baseline global viscoelastic profile. Few studies have evaluated their performance for assessing antiplatelet therapy. They reported conflicting results with a substantial intra- and inter-individual variability (186, 193–195). Each of these POC tests has strengths and weaknesses and no gold standard method for clinical application is yet identified, standardized and validated (196–198).

## Conclusion

The worldwide increasing trend in clinical practice toward patient-centered precision medicine applies to antiplatelet therapy. It aims to select the appropriate antiplatelet agent with the optimal dose and therapy duration and requires careful balancing of benefits and risks in light of each patient's clinical characteristic and circumstances. Despite major advances in antiplatelet therapy, many areas of development deserve further investigation in order to appropriately manage the currently available agents and provide better guidance in clinical scenarios such as bleeding and surgery. Development of effective and safe reversal compounds for antiplatelet agents is also an area of unmet need. Moreover, novel antiplatelet drugs are in the pipeline (among which RUC-4, selatogrel, revacept, glenzocimab; non-exhaustive list) (199–203). How these potential new therapeutics will fit within the current paradigm of antiplatelet therapy and whether they will lead to safer combinations in the clinical practice remain to be determined. Therefore, while substantial research has allowed breakthroughs optimization of antiplatelet therapy, there is still much further to go.

## Author Contributions

GJ and PG designed and drafted the work. AG, ML, and GM-G substantially revised the work. All authors have read and approved the submitted version.

## Conflict of Interest

AG reports honoraria and travel fees from Bayer-Healthcare, Boehringer-Ingelheim, Bristol-Myers-Squibb/Pfizer, LFB, Octapharma, and Sanofi. ML has received speaker fees from Bayer; has participated in industry-funded trials from Idorsia; has served on advisory boards for Servier and JAMP/Orimed Pharma; and has received in-kind and financial support for investigator-initiated grants from Leo Pharma, Roche Diagnostics, Aggredyne, and Fujimori Kogyo. GM-G reports speaker fees and advisory board fees from JAMP Pharma, and research funding from Bayer. The remaining authors declare that the research was conducted in the absence of any commercial or financial relationships that could be construed as a potential conflict of interest.

## Publisher's Note

All claims expressed in this article are solely those of the authors and do not necessarily represent those of their affiliated organizations, or those of the publisher, the editors and the reviewers. Any product that may be evaluated in this article, or claim that may be made by its manufacturer, is not guaranteed or endorsed by the publisher.
